# Selective decontamination regimens in French ICUs: association with reduced infection and resistance emergence

**DOI:** 10.1186/s13613-025-01465-9

**Published:** 2025-03-25

**Authors:** Nicolas Massart, Marc Leone, Florian Reizine, Gary Duclos, Anaïs Machut, Charles-Hervé Vacheron, Anne Savey, Emmanuelle Hammad, Arnaud Friggeri, Alain Lepape, the REAREZO study group

**Affiliations:** 1 Service de Réanimation Polyvalente, Centre Hospitalier de Saint Brieuc, CH de St BRIEUC, 10, Rue Marcel Proust, 22000 Saint-Brieuc, France; 2 Aix Marseille Université, Assistance Publique-Hôpitaux de Marseille, Service d’Anesthésie et de Réanimation, Hôpital Nord, Marseille, France; 3 Service de Réanimation polyvalente, Centre Hospitalier de Vannes, Vannes, France; 4 REA-REZO Infections et Antibiorésistance en Réanimation, Hôpital Henry Gabrielle, Saint-Genis, France; 5 Département d’Anesthésie Médecine Intensive Réanimation, Centre Hospitalier Lyon Sud, Hospices Civils de Lyon, 165 Chemin du Grand Revoyet, 69310 Pierre-Bénite, France; 6 PHE3ID, Centre International de Recherche en Infectiologie, Institut National de la Santé et de la Recherche Médicale U1111, CNRS Unité Mixte de Recherche 5308, École Nationale Supérieure de Lyon, Université Claude Bernard Lyon 1, Villeurbanne, France; 7 MEPHI, IHU Méditerranée Infection, Aix-Marseille Université, Assistance Publique-Hôpitaux de Marseille, IHU Méditerranée Infection, Marseille, France

**Keywords:** Bacteremia, Pneumonia, Colonization, Acquired infection, Selective decontamination of the digestive tract

## Abstract

**Background:**

Despite randomized controlled trials with favorable results, few intensive care units (ICUs) implemented selective decontamination (SD) for ICU-acquired infection prevention. We aimed to evaluate, SD implementation and associated-effects in a large network of French ICUs.

**Methods:**

This study was conducted using the healthcare-associated infection surveillance cohort “REA-REZO” involving 193 participating ICUs. All patients receiving invasive mechanical ventilation for > 24 h were included. In addition to standard of care (SOC), six ICUs applied a SD strategy during the study period. The primary endpoint was the rate of ICU-acquired infection. A propensity-score matched analysis was conducted using non-parsimonious regression model. The secondary endpoint was the rate of colonization by multidrug resistant organisms (MDRO) during the ICU stay.

**Results:**

Among 81,661 patients with invasive mechanical ventilation for longer than 24 h, 2727 patients receiving SD were matched with 2 727 receiving SOC. The ICU-acquired infection incidence was lower in the SD group as compared with the SOC group (Incidence Rate Ratio = 0.66 [0.60–0.73]; p < 0.001) although the ICU mortality was similar (31.9% vs 32.5%, respectively p = 0.689). Acquisition of MDRO was lower in the SD group than in the SOC group (40 (1.5%) patients vs. 139 (5.1%) patients p < 0.001).

**Conclusions:**

These results showed that a strategy of SD was associated with reduced ICU-acquired infection incidence and decreased emergence of MDRO, while the mortality was not affected.

**Supplementary Information:**

The online version contains supplementary material available at 10.1186/s13613-025-01465-9.

## Background

Among intensive care units (ICUs) acquired infection prevention methods, selective decontamination (SD) is a major intervention associated with a decrease in ICU-acquired infection incidence [1–3]. It consists of the administration of oropharyngeal or digestive antibiotics targeting *Enterobacterales* and non-fermenting Gram-negative bacilli, while preserving associated anaerobic flora, combined with a short course of intravenous antibiotics targeting pathogens responsible for early onset pneumonia. For decades now, various SD regimens have been assessed, either restricted to the oropharyngeal tract, or to the digestive tract in association or not with a 1 to 5 days various systemic antibiotics or with cutaneous decontamination [2–7].

Despite these promising findings, fear of antimicrobial resistant selection raised reluctance against it and only few ICUs implemented this strategy [8, 9]. In France, the use of a SD strategy is supported by the French recommendations for the prevention of ventilator-associated pneumonia (VAP) [10] but it remains scarcely implemented [11]. Similarly, SD implementation was controversial in the United Kingdom [12–14]. The major reason for not implementing SD was related to the fear of emergence of multidrug resistant organisms (MDRO) [12–14]. However, recent studies and meta-analysis suggested a protective effect on the level of resistance instead of a boosting effect [3].

As this prophylaxis remains a matter of debates, we aimed to evaluate, though a large nationwide cohort study, SD implementation and related effects in a network of French ICUs.

## Methods

### Patients and setting

This study was conducted using the REA-REZO prospective continuous multicenter cohort. It is a healthcare-associated infection surveillance network with collection of patient-level data of all adult patients hospitalized for at least 2 calendar days in any of the 193 contributing ICUs of the REA-REZO network since January 2018. Surveillance focuses on ICU-ICU-acquired infection and was discontinued when patients either died or were discharged from ICU. The detailed protocol for data collection and monitoring is available at: https://rearezo.chu-lyon.fr/.

In the present study, all patients admitted to a participating ICU from 1st January 2018 to 31th December 2022 were screened, but only those receiving invasive mechanical ventilation for > 24 h were included.

### Definition

Infection was considered as ICU-acquired if diagnosed 48 h after ICU admission and not incubating on admission [14]. The ICU-acquired infections were diagnosed by the treating physicians. Bloodstream infection (BSI) was defined as a positive blood sample occurring 48 h or more after admission. Regarding common skin contaminants, 2 positive blood cultures drawn on separate occasions were required [15]. The diagnosis of VAP was considered in patients receiving invasive mechanical ventilation for 48 h or more and until 48 h after tracheal extubation, based on clinical signs (fever, purulent sputum, hypoxia), radiological findings (new infiltrate), and leukocytosis [10]. Microorganisms responsible for infection were considered as MDRO according to the European Society of Clinical Microbiology and Infectious Disease definition [16].

A rectal colonization surveillance was conducted in 103 out of 193 participating ICUs. In these ICUs, the patients were screened for MDRO rectal carriage on rectal swabs at ICU admission, weekly afterwards and at discharge. As described elsewhere, the patients with no prior colonization (no colonization at admission) who were tested positive for MDRO on either rectal screening or on a blood or respiratory sample were considered as having MDRO acquisition [17]. Analyses comparing MDRO colonization were restricted to patients admitted to an ICU with systematic rectal colonization surveillance.

Systemic antibiotic at ICU admission was defined as a curative antibiotic for a suspected or confirmed infection within 24 h before or after admission. Workload in participating ICUs was extrapolated as follow: number of admissions by year /number of ICU bed in the unit of admission.

Hydro-alcoholic solution (HAS) consumption (L/1 000 patient-days/year) was used as an indicator of hygiene quality. The HAS consumption was calculated with the consumption of HAS in one year divided by the number of days of hospitalization in the same ICU within the same year.

### Intervention

In addition to standard of care (SOC) [10], six ICUs applied a SD strategy during the study period. Of them, four ICUs that did not initially apply SD in intubated patients started to applied SD protocol during the study period, while another that applied a SD strategy discontinued its use and no patients received it in the unit thereafter. The periods of implementation are reported in Supplementary Table 1. The SD regimens differed between the six ICUs. Only two ICUs applied a regimen that included the use of a systemic intravenous antibiotic (either cefotaxime or cefazolin) in addition to topical antibiotics. Two other ICUs only applied topical antibiotics without systemic antibiotic nor cutaneous decontamination. Finally, the last two ICUs applied a multiple site decontamination (MSD) consisting of the administration of topical antibiotics 4 times daily in the oropharynx and the gastric tube, 4% chlorhexidine body-wash once daily and a 5-day nasal mupirocin course, without systemic antibiotic course (Supplementary Table 1).

Selective decontamination was used to prevent acquired infections in all patients expected to be intubated for more than 24 h, continuing for the entire duration of intubation. Eligible patients were divided into two groups: the SD group if they were admitted to an ICU applying a decontamination regimen and the SOC group in ICUs not applying a decontamination regimen.

### Primary and secondary endpoints

The primary endpoint was the rate of ICU-acquired infection, while the secondary endpoints were VAP and the BSI incidence rates. The ICU mortality, length of invasive mechanical ventilation, number of invasive mechanical ventilation free days, duration of ICU stay, and ICU free days were also collected. Finally, we assessed the proportion of patients with MDRO acquisition, but this analysis was restricted to patients admitted to an ICU using systematic rectal colonization surveillance.

The ICU and mechanical ventilation-free days were composite outcomes, which combined survival and ICU duration or survival and length of invasive mechanical ventilation. The numbers of ICU-free days or ventilatory-free days were calculated as 28 minus the number of days in ICU or with mechanical ventilator support during the first 28 days after admission. Patients who died within 28 days after admission were assigned the worst possible outcome of zero ICU-free days or ventilatory-free days.

### Statistical analysis

Statistical analysis was performed with the statistical software R 4.1.1. Categorical variables were expressed as percentages and continuous variables as median and interquartile range (IQR). The chi-square test and Fisher exact test were used to compare categorical variables and the Man-Whitney U test or the Wilcoxon for continuous variables.

To draw unbiased marginal estimates of exposure effect, a propensity-score matched analysis was performed to evaluate the impact of SD regimen on outcome. Propensity score was calculated using non-parsimonious logistic regression model including all available baseline characteristics [including type of ICU of admission, ICU workload, HAS consumption, age, sex, immunodepression, simplified acute physiology score (SAPS) II, year of admission, reason for admission, localization before admission, COVID-19 status, early management] and corresponds for each patient to his or her probability to be admitted to an ICU where SD has already been implemented. Using the “MatchIt” package, a k-nearest neighbor algorithm was used for propensity-score matching with a 1:1 ratio. The balance between matched groups was evaluated by the analysis of the standardized mean differences after matching. A post-matching difference < 0.1 was considered as an optimal bias reduction.

Kaplan–Meier survival curves with log-rank test were used for survival analysis. Incidence rates were compared using a Poisson regression model. Multivariable analysis was performed in complete cases. All tests were two-sided, and p < 0.05 was considered statistically significant.

## Results

### Patients

**Fig. 1:**
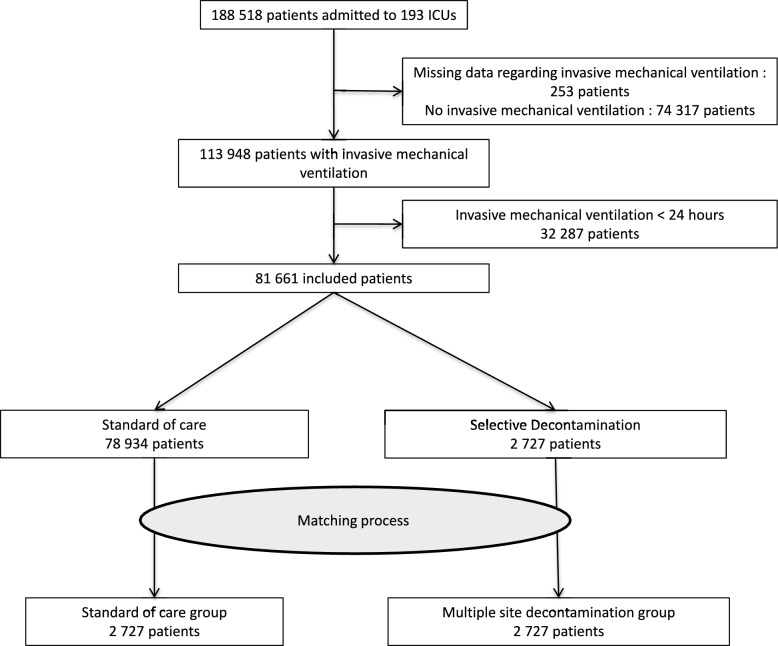
Flow chart

During the study period, 188,518 patients were admitted to the 193 participating ICUs. Among them, 113,948 patients received invasive mechanical ventilation and 81,661 received it for longer than 24 h and were included in the present analysis. Of the 193 participating ICUs, only six (3.1%) applied a SD regimen corresponding to 2727 (3.4%) patients (Fig. 1). The SD group and the SOC group had a similar workload (29.8 admission per bed per year [27.4–32.6] vs 27.5 [22.1–33.2] respectively, p = 0.377) and a similar HAS consumption (0.13 L/patient/day [0.10–0.14] vs 0.15 [0.11–0.15] p = 0.357). The MDRO prevalence rate on admission rectal swab was lower in the SD group as compared with the SOC group (1.2% [1.1–2.0] vs 5.0% [1.7–7.5]; p < 0.001). Details on the features of patients are available in Supplementary Table 2. Propensity score matching resulted in a well-balanced study population that included 2272 patients in both arms (Table 1).

**Table 1:** Baseline characteristics of study patients (matched patient pairs)

Variables	Standard of Care	Selective Decontamination	SMD
	n = 2727	n = 2727	
Type of ICU of admission Polyvalent (vs surgical)—n (%)	2535 (93.0)	2522 (92.5)	0.02
Workload in ICU of admission, number of admissions per bed per year	28.85 [25.56–36.97]	32.60 [26.50–33.25]	0.05
Hydro-alcoholic solution consumption in ICU of admission, L per patient-day	0.15 [0.14–0.20]	0.16 [0.13–0.18]	0.05
Age, year	65.60 [53.95–74.20]	63.30 [51.20–71.90]	0.09
Male—n (%)	1859 (68.2)	1862 (68.3)	0.00
Immunocompromised			0.04
Neutropenia < 500 G/L—n (%)	124 (4.5)	141 (5.2)	0.04
Others—n (%)	212 (7.8)	182 (6.7)	0.16
None—n (%)	2391 (87.7)	2404 (88.2)	0.07
SAPS II	54 [41–67]	54 [40–68]	0.03
Year of admission			
2018—n (%)	282 (10.3)	437 (16.0)	0.15
2019—n (%)	343 (12.6)	360 (13.2)	0.02
2020—n (%)	222 (8.1)	285 (10.5)	0.07
2021—n (%)	756 (27.7)	570 (20.9)	0.17
2022—n (%)	1124 (41.2)	1075 (39.4)	0.04
Localization before admission			
Home—n (%)	1692 (62.0)	1768 (64.8)	0.06
Acute care ward—n (%)	912 (33.4)	830 (30.4)	0.07
Other ICU—n (%)	123 (4.5)	129 (4.7)	0.01
Type of admission			
Planned surgery—n (%)	152 (5.6)	149 (5.5)	0.00
Urgent surgery—n (%)	873 (32.0)	932 (34.2)	0.04
Medicine—n (%)	1702 (62.4)	1646 (60.4)	0.04
Trauma—n (%)	598 (21.9)	690 (25.3)	0.08
COVID-19—n (%)	313 (11.5)	264 (9.7)	0.06
Early management			
Therapeutic antibiotics—n (%)	1411 (51.7)	1409 (51.7)	0.00
Central venous catheter—n (%)	2483 (91.1)	2490 (91.3)	0.01

Categorical variables are expressed as percentages and continuous variables as median and interquartile range

*ICU* Intensive-care unit, *COVID-19* SARS-COV 2 associated infection disease, *SMD* standardized mean difference

### Outcomes

**Fig. 2:**
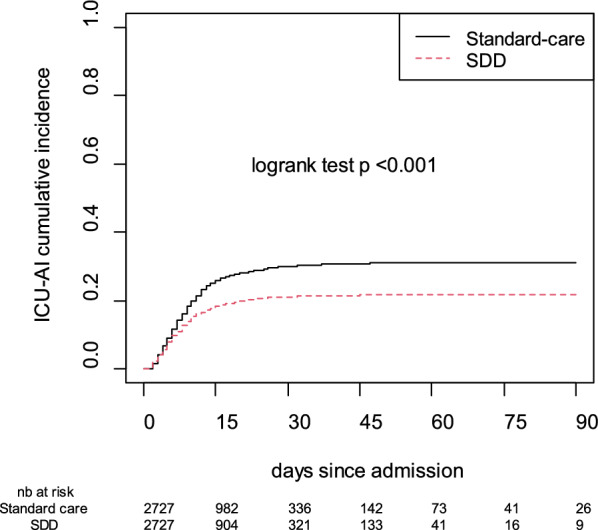
AI incidence in both groups (matched patients pairs)

During the study period, we identified 678 ICU-acquired infections including 483 VAP and 195 BSI in 511 (18.7%) patients in the SD group and 1,087 ICU-acquired infections including 792 VAP and 295 BSI in 724 (26.5%) patients in the SOC group (p < 0.001). Similarly, less patients in the SD group developed VAP (14.7%) and BSI (6.4%), as compared with the SOC group (22.2%, and 9.6%, respectively, both p < 0.001) (Table 2 and Fig. 2).

**Table 2:** Outcomes (matched patient pairs)

Variables	Standard of Care	Selective Decontamination	p-value
	n = 2727	n = 2727	
Length of stay in ICU, days	10 [5–19]	10 [5–18]	0.159
ICU-free days at day 28	0 [0–10]	0 [0–12]	0.716
Length of mechanical ventilation, days	6 [3–14]	6 [3–12]	0.049
Ventilator-free days at day 28	15 [0–24]	16 [0–24]	0.087
Death in the ICU—n (%)	886 (32.5)	868 (31.9)	0.689
Acquired infections	724 (26.5)	511 (18.7)	< 0.001
Bloodstream infection—n (%)	261 (9.6)	174 (6.4)	< 0.001
Pneumonia—n (%)	604 (22.2)	401 (14.7)	< 0.001
Time to first acquired infection, days	8 [5–13]	7 [4–10]	< 0.001
MDRO acquisition*^£^—n (%)	139 (5.1)	40 (1.5)	< 0.001
MDRO colonization acquisition^$^—n (%)	109 (5.2)	23 (1.2)	< 0.001
AI due to MDRO^£^—n (%)	43 (1.6)	20 (0.7)	0.005

Categorical variables are expressed as percentages and continuous variables as median and interquartile range

*ICU* Intensive-care unit, *MDRO* Multi Drug Resistant Micro Organisms,

^*^Reflect both rectal colonization acquisition and AI due to MDRO with some patients acquiring bot MDRO rectal colonization and AI due to MDRO

^£^The variable was assessed among the 2727/2727 patients of the standard of care and selective decontamination group

^$^The variable was evaluated only in patients in whom MDRO rectal colonization was assessed (admission in an ICU that systematically performed rectal sample for MDRO detection as part of daily care), corresponding to 1930 patients of the selective decontamination group and 2102 patients of the standard of care group

During the study period, 27,723 invasive mechanical ventilation days and 39,526 ICU days were collected in the SD group and 31,159 invasive mechanical ventilation days and 42,361 ICU days in the SOC group. The incidence of ICU-acquired infection was 17 per 1000 patient days in the SD group versus 26 AI per 1000 patient days in the SOC group (Incidence Rate Ratio [IRR] = 0.66 [0.60–0.73] p < 0.001). Similarly, the BSI incidence was 5 per 1000 patient days as compared with 7 per 1000 patient days (IRR = 0.70 [0.57–0.84] p < 0.001) and the incidence of VAP was 17 per 1000 invasive mechanical ventilation days and 25 per 1000 invasive mechanical ventilation days in the SD group and the SOC group, respectively (IRR = 0.68 [0.61–0.76] p < 0.001).

The patients in the SD group had a small, approaching statistically significant, shorter exposure to invasive mechanical ventilation than those in the SOC group (6 days [3–12] vs 6 days [3–14], respectively, p = 0.049). The length of stay and ICU mortality were similar in both groups (Table 3 and Supplementary Fig. 1).

**Table 3:** Data regarding acquired infections (matched patient pairs)

Variables	Standard of Care	Selective Decontamination	p-value
	n = 1087	n = 678	
Site of ICU-acquired infections*			0.966
Ventilator-associated pneumonia vs Bloodstream infection– no. (%)	797 (71.0)	483 (70.8)	
ICU-acquired infection due to a MDRO—no. (%)	89 (7.9)	27 (4.0)	0.001
ESBL-PE– no. (%)	61 (68.5)	9 (33.3)	
Carbapenem resistant *Enterobacterales*—no. (%)	6 (6.7)	2 (7.4)	
Vancomycin resistant *Enterococcus sp.*—no. (%)	0 (0.0)	1 (3.7)	
MDR NF-GNB—no. (%)	10 (11.2)	5 (18.5)	
Methicillin Resistant *Staphylococcus aureus*– no. (%)	12 (13.5)	10 (37.0)	

Bloodstream infection	n = 325	n = 199	
Micro-organisms responsible for first BSI			< 0.001
*Enterobacterales*—no. (%)	119 (36.6)	69 (34.7)	
Non-fermenting Gram negative Bacilli—no. (%)	38 (11.7)	15 (7.5)	
*Staphylococcus aureus*—no. (%)	40 (12.3)	20 (10.1)	
Coagulase negative *Staphylococcus*^$^—no. (%)	42 (12.9)	39 (19.6)	
*Streptococcus sp*.—no. (%)	16 (4.9)	11 (5.5)	
*Enterococcus sp*—no. (%)	27 (8.3)	22 (11.1)	
*Candida sp.*—no. (%)	32 (9.8)	8 (4.0)	
Virus—no. (%)	1 (0.3)	2 (1.0)	
Others—no. (%)	3 (0.9)	8 (4.0)	
Anaerobes—no. (%)	6 (1.8)	3 (1.5)	
Polymicrobial BSI—no. (%)	51 (15.7)	30 (15.1)	0.948
Source of BSI			0.005
Digestive tract– no. (%)	39 (12.9)	34 (17.3)	
Urinary tract—no. (%)	18 (5.9)	9 (4.6)	
Other—no. (%)	84 (27.7)	61 (31.1)	
Catheter—no. (%)	82 (27.1)	52 (26.5)	
Skin and soft tissue—no. (%)	11 (3.6)	10 (5.1)	
Pulmonary—no. (%)	68 (22.4)	23 (11.7)	

Pneumonia	n = 797	n = 483	
Micro-organisms responsible for first Pneumonia			< 0.001
*Enterobacterales*—no. (%)	354 (44.4)	166 (34.4)	
Non-fermenting Gram negative Bacilli—no. (%)	175 (22.0)	65 (13.5)	
*Staphylococcus aureus*—no. (%)	134 (16.8)	97 (20.1)	
Coagulase negative *Staphylococcus*—no. (%)	4 (0.5)	19 (3.9)	
*Streptococcus sp*.—no. (%)	34 (4.3)	31 (6.4)	
*Enterococcus sp*—no. (%)	11 (1.4)	11 (2.3)	
Virus—no. (%)	2 (0.3)	11 (2.3)	
Filamentous fungi—no. (%)	11 (1.4)	9 (1.9)	
Others ^¤^– no. (%)	62 (7.8)	72 (14.9)	
Anaerobes—no. (%)	1 (0.1)	1 (0.2)	
Polymicrobial Pneumonia—no. (%)	253 (31.7)	134 (27.7)	0.148

Categorical variables are expressed as percentages and continuous variables as median and interquartile range

*ICU* Intensive-care unit. *ESBL-PE* extended spectrum beta lactamase producing *Enterobacterales*, *MDRO* multi drug resistant micro organisms

^*^Since patients may acquired more than one ICU-acquired infection, there is more ICU- acquired infection than are patients with ICU- acquired infection

^$^Regarding coagulase negative Staphylococci (and other common skin contaminant), 2 positives blood cultures drawn on separate occasions were required

^¤^ Among patients with Pneumonia caused by “other” microorganisms, 9 had a pulmonary sample positive for *Candida sp.*, 1 in the SD group and 8 in the SC group

### Microbiology

Data on organisms responsible for ICU-acquired infections are reported in Table 3. *Enterobacterales* represented 40.0% of ICU-acquired infections followed by non-fermenting Gram-negative bacilli (16.6%) and *Staphylococcus aureus* (16.5%). The distribution of organisms responsible for ICU-acquired infections differed between groups for both BSI (p < 0.001) and pneumonia (p < 0.001). Specifically, a lower proportion of *Enterobacterales* (34.4% vs 44.4% p < 0.001) and non-fermenting Gram-negative bacilli (13.5% vs 22.0% p < 0.001) was observed in the pneumonia of the SD group as compared with the SOC group. A higher proportion of coagulase negative *Staphylococcus* was observed in BSI cases of the SD group (19.9% vs 12.9%, respectively, p = 0.040). The sources of BSI differed between groups (p < 0.001) since less VAP were reported in the SD group (11.7% vs 22.4%, p < 0.001). In contrast, a higher proportion of other sources was found in this group (34.7% vs 28.0%, p < 0.001).

Regarding MDRO acquisition, 40 (1.5%) patients of the SD group acquired MDRO (23 acquired MDRO rectal colonization/20 had an AI due to MDRO/3 had both) as compared with 139 (5.1%) patients in the SOC group (109 acquired MDRO rectal colonization/43 had an AI due to MDRO/13 had both) (p < 0.001). In the SOC group, the primary mechanism of bacterial resistance was the production of extended-spectrum beta-lactamase by Enterobacterales, whereas in the SD group, methicillin resistant *Staphylococcus aureus* was the predominant MDRO. (Table 3).

## Discussion

In this nationwide cohort study, we observed that the implementation of SD remains rare in this network of ICUs and that various regimens are used. The patients admitted to ICUs applying SD had a lower incidence of ICU-acquired infections although this decrease did not translate into lower mortality rate. Finally, in contrast to old beliefs, the rate of MDRO was decreased in the patients receiving SD, as compared with SOC.

The low rate of implementation of SD regimen has been previously reported [11]. In a Dutch survey, Elderman et al. reported that factor associated with SD implementation were related to ICU organization such as numbers of beds per ICU, number of full-time equivalent intensivists and nurses. Regional differences were also notified, mostly based on the influence of local experts [7]. In a survey conducted in the UK, Shah et al. reported a low implementation rate, due to the fear of increased antimicrobial resistance but also related to technical aspects such as disinterest of the drug companies [18].

Finally, in a European survey, Miranda et al. observed a low implementation rate of SD of only 17%, which was not related to the incidence of MDRO or with a lack of belief in the validity of the cluster randomized design of the studies in support of SD [19].

Another interesting finding is the heterogeneity in decontamination regimens, which were irregularly combined with either intravenous antibiotics or chlorhexidine body washes. To date, few studies compared SD regimens with each other. In two previous RCTs, De Smet et al. and Oostdijk et al. observed that SDD (in association with a short course of intravenous antibiotic) was superior to SOD for AI prevention but also for preventing death in the ICU [2, 20]. In another randomized controlled trial (RCT), Camus et al. observed that the association of topical antibiotic (without intravenous prophylaxis) with chlorhexidine bodywash and intranasal mupirocin was synergic and superior to topical antibiotic administration alone [3]. Future studies are needed to better identify best available composition of decontamination strategy.

The incidences of ICU-acquired infections that were reported here are in line with those reported in previous studies looking at the VAP incidence ranging from 5 to 40% and the BSI incidence ranging from 2 to 5% [21–23]. However, the decrease in ICU-acquired infections using SD was lower than previously reported. In a recent meta-analysis of randomized controlled trial, Hammont et al. reported a relative risk of 0.44 for VAP and of 0.68 for BSI with this prophylaxis [3]. The diagnosis of VAP relies on the clinician interpretation and we have to acknowledge significant differences in the method for VAP diagnosis between centers [24–26]. Conversely, BSI diagnosis is more consensual. In our study, the BSI incidence was also decreased, confirming results of studies conducted elsewhere [2, 5, 25]. Interestingly, BSI incidence was not very different from those observed in study conducted in France in the early 2000 [27]. However, the epidemiology differed with a lower representation of coagulase negative Staphylococci in the present report.

Our decreased risk of MDRO acquisition with SD regimens is consistent with previous studies and meta-analysis [3, 27–29]. This finding is of utmost importance since the detractors to SD stresses on the fear of MDRO emergence to limit the diffusion of SD. Our study confirms the favorable safety profile of a decontamination strategy with decreased prevalence of MDRO in the treated group. In fact, the emergence of MDRO is multifactorial and probably depends on the global use of antibiotics during the entire patient hospital stay. Reducing the number of infections should result in a decreased use of antibiotics, and resistances per se. In brief, the prevention of ICU-acquired infections leads to a decrease of antimicrobial agent consumption, itself decreasing antimicrobial resistance selection pressure [6].

## Limitations

This study was not interventional, and the two groups of ICUs probably differed for several and unmeasured practices. As underlined above, the methods for diagnosis, even if relying on national guidelines, are variable from one site to another. Reliability of diagnosis methods are different between SD and SOC due to the impact of local antibiotics in SD cohort. However, even though VAP diagnosis could be considered as subjective, BSI diagnosis is less questionable. Second, the regimen was used in the six ICUs only, thereby limiting external validity. Moreover, there were dissimilar among the regimens consisting of topical and systemic antibiotics or only topical antibiotics. In addition, few of them introduced skin disinfection. In a systematic review, Roquilly et al. showed that only regimens based on topical and systemic use of antibiotics were associated with decreased mortality rates. Then, we did not measure the rate of resistance in the different units, while there is still a doubt about the use of SD in environment in which there is a high rate of multidrug resistance. Finally, uncontrolled variables may also have affected our findings.

## Conclusions

Our observational study suggested an association between the ICU using SD and lower rates of ICU-acquired infections, notably VAP and BSI. Importantly, MDRO acquisition rate was lower among patients receiving SD. However, this association did not translate into a decrease in mortality. These results, however, tend to confirm that SD may be associated with improved outcome and decreased emergence of MDRO, reinforcing the need for a randomized controlled trial.

## Supplementary Information

Additional file1

## Data Availability

The datasets generated during the current study are available from the corresponding author on reasonable request.
