# Supplementary material for: Selective decontamination regimens in French ICUs: association with reduced infection and resistance emergence
Source: Ann Intensive Care. 2025 Mar 25;15:41. doi: 10.1186/s13613-025-01465-9 (PMC11936853; doi:10.1186/s13613-025-01465-9)
Supplement: Supplementary file 1 — Additional file1 [file 13613_2025_1465_MOESM1_ESM.docx]

**Supplementary Table 1. Characteristics of the selective digestive decontamination treatments in the different ICUs**

| **Hospital centers** | **Components of SDD** | **Frequency** | **Patients receiving SDD** | **Period of implementation** |
| --- | --- | --- | --- | --- |
| ICU of the Saint-Brieuc hospital center | Gentamycin 543 mg  Enteral amphotericin B 2g  Colistin 400mg  4% chlorhexidine body-wash once daily  5-day nasal mupirocin course | QID | Patients receiving mechanical ventilation during at least 48 hours | Implementation since May 1^st^ 2021 |
| ICU of the Vannes hospital center | Gentamycin 543 mg  Enteral amphotericin B 2g  Colistin 400mg  4% chlorhexidine body-wash once daily  5-day nasal mupirocin course | QID | Patients receiving mechanical ventilation during at least 48 hours | Implementation since June 1^st^ 2021 |
| Surgical ICU of the Marseille Nord academic hospital center | Tobramycine 80 mg  Enteral amphotericin B 500mg Colistin 100mG  Intravenous Cefazolin 2G TID during 1 day | TID | Patients receiving mechanical ventilation | Implementation before 2018, no interruption |
| ICU of the Annecy-Genevois hospital center | Gentamycin 543 mg  Enteral amphotericin B 2g  Colistin 400mg  Intravenous Cefotaxime 1g QID during 4 days | QID | Patients receiving mechanical ventilation during at least 48 hours | Implementation since July 1^st^ 2021 |
| ICU of the Quimper hospital center | Gentamycin 543 mg  Enteral amphotericin B 2g  Colistin 400mg | QID | Patients receiving mechanical ventilation during at least 48 hours | Implementation since October 11^th^ 2021 |
| Surgical ICU of the Nimes academic hospital center | Tobramycine 80 mg  Enteral amphotericin B 500mg Colistin 100mG | QID | Patients receiving mechanical ventilation | Implementation before 2018  Interruption of decontamination strategy since March 1^st^ 2020 |

Notes. QID: *Quater in die*; TID: *Ter in die*

**Supplementary Table 2. Baseline characteristics, full population.**

|  | Complete population | Standard-Care | Selective Decontamination | p-value |
| --- | --- | --- | --- | --- |
| Variables | n = 81 661 | n = 78 934 | n = 2 727 |  |
| Type of ICU |  |  |  | <0.001 |
| Burn – n (%) | 10 (0.0) | 10 (0.0) | 0 (0.0) |  |
| Cardiac – n (%) | 581 (0.7) | 581 (0.7) | 0 (0.0) |  |
| Surgical – n (%) | 8911 (10.9) | 8706 (11.1) | 205 (7.5) |  |
| Medical – n (%) | 11586 (14.2) | 11586 (14.7) | 0 (0.0) |  |
| Neurology – n (%) | 1456 (1.8) | 1456 (1.9) | 0 (0.0) |  |
| Medico-surgical – n (%) | 58859 (72.3) | 56337 (71.6) | 2522 (92.5) |  |
| Workload, number of admissions per bed per year | 28.60 [23.50-33.31] | 28.60 [23.20-33.31] | 32.60 [26.50-33.25] | <0.001 |
| Hydro-Alcoholic Consumption, L per patient-day | 0.13 [0.09-0.15] | 0.13 [0.09-0.15] | 0.16 [0.13-0.18] | <0.001 |
| Age, year | 66.30 [55.80-74.30] | 66.40 [56.00-74.40] | 63.30 [51.20-71.90] | <0.001 |
| Male – n (%) | 53755 (65.8) | 51894 (65.7) | 1861 (68.3) | 0.007 |
| Immunocompromised |  |  |  | <0.001 |
| Neutropenia < 500 G/L – n (%) | 1310 (1.6) | 1169 (1.5) | 141 (5.2) |  |
| Others – n (%) | 10286 (12.6) | 10104 (12.8) | 182 (6.7) |  |
| None – n (%) | 70065 (85.8) | 67661 (85.7) | 2404 (88.2) |  |
| SAPS II | 54.00 [41.00-68.00] | 54.00 [41.00-68.00] | 54.00 [40.00-68.00] | 0.583 |
| Year of admission |  |  |  | <0.001 |
| 2018 – n (%) | 25950 (31.8) | 25513 (32.3) | 437 (16.0) |  |
| 2019 – n (%) | 16286 (19.9) | 15926 (20.2) | 360 (13.2) |  |
| 2020 – n (%) | 13964 (17.1) | 13679 (17.3) | 285 (10.5) |  |
| 2021 – n (%) | 13807 (16.9) | 13237 (16.8) | 570 (20.9) |  |
| 2022 – n (%) | 11654 (14.3) | 10579 (13.4) | 1075 (39.4) |  |
| Localization before admission |  |  |  | <0.001 |
| Home – n (%) | 46043 (56.5) | 44282 (56.2) | 1761 (64.7) |  |
| Acute care Ward – n (%) | 29839 (36.6) | 29009 (36.8) | 830 (30.5) |  |
| Other ICU – n (%) | 5612 (6.9) | 5483 (7.0) | 129 (4.7) |  |
| Type of admission |  |  |  | <0.001 |
| Planned surgery – n (%) | 4136 (5.1) | 3987 (5.1) | 149 (5.5) |  |
| Urgent surgery – n (%) | 15795 (19.4) | 14863 (18.8) | 932 (34.3) |  |
| Medicine – n (%) | 61659 (75.6) | 60022 (76.1) | 1637 (60.2) |  |
| Trauma – n (%) | 6419 (7.9) | 5729 (7.3) | 690 (25.4) | <0.001 |
| COVID-19 – n (%) | 8150 (10.1) | 7886 (10.1) | 264 (9.8) | 0.645 |
| Early management |  |  |  |  |
| Therapeutic antibiotics – n (%) | 54343 (67.0) | 52945 (67.5) | 1398 (51.5) | <0.001 |
| Central venous catheter – n (%) | 73571 (90.2) | 71082 (90.2) | 2489 (91.3) | 0.050 |

Notes. ICU : Intensive-care unit. AI : Acquired Infection. COVID-19 : SARS-COV 2 associated infection disease.

**Supplementary Table 3. Outcomes in full population.**

|  | Complete population | Standard-Care | Selective Decontamination | p-value |
| --- | --- | --- | --- | --- |
| Variables | n = 81 661 | n = 78 934 | n = 2 727 |  |
| Length of stay in ICU, days | 10.00 [5.00, 19.00] | 10.00 [5.00, 19.00] | 10.00 [5.00, 18.00] | 0.032 |
| Length of mechanical ventilation, days | 7.00 [3.00, 14.00] | 7.00 [3.00, 14.00] | 6.00 [3.00, 12.00] | <0.001 |
| Death in the ICU – n (%) | 26483 (32.5) | 25615 (32.5) | 868 (31.9) | 0.574 |
| Acquired infection | 19470 (23.8) | 18959 (24.0) | 511 (18.7) | <0.001 |
| Bloodstream infection – n (%) | 6459 (7.9) | 6286 (8.0) | 174 (6.4) | 0.002 |
| Pneumonia – n (%) | 16608 (20.3) | 16207 (20.5) | 401 (14.7) | <0.001 |
| MDRO colonization acquisition – n (%) | 2098 (2.6) | 2075 (2.6) | 23 (0.8) | <0.001 |

Notes. ICU : Intensive-care unit. AI : Acquired Infection. MDRO : Multi Drug Resistant Micro Organisms.

**Supplementary Figure 1. Survival curves**
